# Oxytocin Administration Alleviates Acute but Not Chronic Leptin Resistance of Diet-Induced Obese Mice

**DOI:** 10.3390/ijms20010088

**Published:** 2018-12-26

**Authors:** Mehdi Labyb, Chloé Chrétien, Aurélie Caillon, Françoise Rohner-Jeanrenaud, Jordi Altirriba

**Affiliations:** 1 Laboratory of Metabolism, Department of Medicine Specialties, Faculty of Medicine, University of Geneva, CH1211 Geneva, Switzerland; El-Mehdi.Labyb@etu.unige.ch (M.L.); Aurelie.Caillon@unige.ch (A.C.); Francoise.Jeanrenaud@unige.ch (F.R.-J.); 2 Department of Cell Physiology and Metabolism, Geneva Medical Centre, CH1211 Geneva, Switzerland; Chloe.chretien21@gmail.com

**Keywords:** oxytocin, leptin, obesity, food intake

## Abstract

Whereas leptin administration only has a negligible effect on the treatment of obesity, it has been demonstrated that its action can be improved by co-administration of leptin and one of its sensitizers. Considering that oxytocin treatment decreases body weight in obese animals and humans, we investigated the effects of oxytocin and leptin cotreatment. First, lean and diet-induced obese (DIO) mice were treated with oxytocin for 2 weeks and we measured the acute leptin response. Second, DIO mice were treated for 2 weeks with saline, oxytocin (50 μg/day), leptin (20 or 40 µg/day) or oxytocin plus leptin. Oxytocin pre-treatment restored a normal acute leptin response, decreasing food intake and body weight gain. Chronic continuous administration of oxytocin or leptin at 40 µg/day decreased body weight in the presence (leptin) or in the absence (oxytocin) of cumulative differences in food intake. Saline or leptin treatment at 20 µg/day had no impact on body weight. Oxytocin and leptin cotreatments had no additional effects compared with single treatments. These results point to the fact that chronic oxytocin treatment improves the acute, but not the chronic leptin response, suggesting that this treatment could be used to improve the short-term satiety effect of leptin.

## 1. Introduction

While oxytocin was historically recognized for its peripheral role in parturition and lactation, we now know that its central release influences various types of behaviors and participates in the regulation of energy balance [1]. Several studies reported that oxytocin administration reduces body fat stores and improves glucose tolerance in various animal models of obesity and in humans [1,2,3,4,5,6,7,8,9,10,11].

Leptin, initially described as a satiety hormone, has a profound impact on central circuits controlling body weight homeostasis, as well as glucose and lipid metabolism [12]. When it was discovered, leptin monotherapy was envisioned to cure the overwhelming worldwide epidemic of obesity and its metabolic comorbidities. Initial results obtained in obese subjects using very high doses of leptin indicated a tendency to decrease body weight. However, such high doses are not applicable for general use and lower concentrations were inefficient [13,14,15,16,17], being only applicable for leptin-deficient subjects [18,19]. Although these data were disappointing, they boosted additional research. One of the approaches consisted of identifying “leptin sensitizer” compounds that could amplify the actions of exogenous leptin [20,21,22]. In the present study, we hypothesized that oxytocin could be such a candidate as: (1) oxytocin administration is able to decrease food intake and body fat gain in animal models of leptin resistance [1,2,3,4,5,6,7,8,9,10,11]; (2) oxytocin-expressing neurons are activated by leptin administration [23]; (3) intracerebroventricular injection of an oxytocin antagonist blunts the anorexigenic effect of leptin [23].

In view of these considerations, our aim was to determine whether chronic oxytocin treatment of obese mice is able to improve leptin sensitivity tested acutely and to overcome leptin resistance during chronic infusion.

## 2. Results

**Figure 1:**
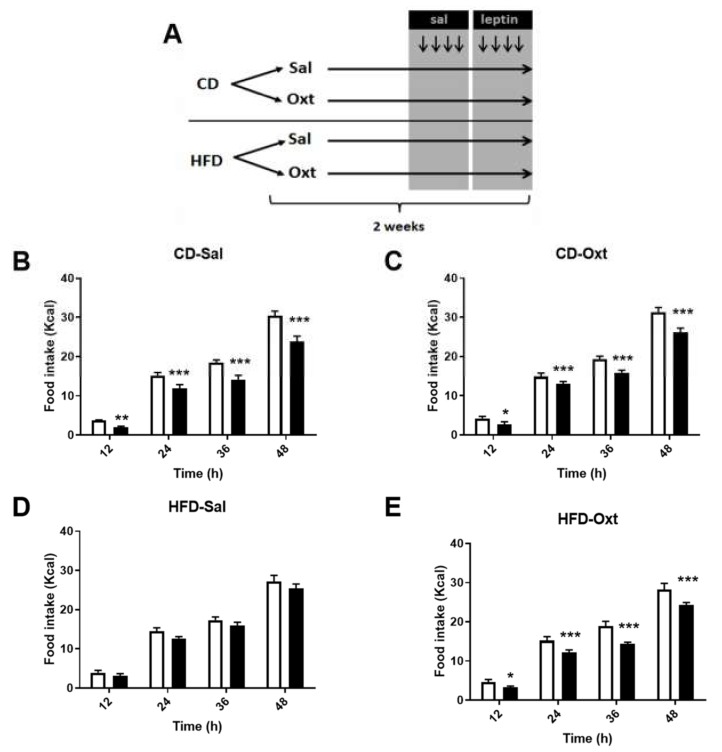
Acute leptin effects on food intake. (**A**) Experimental design described in Material and Methods. Mice were fed a chow diet (CD) or a high fat diet (HFD) during 10 weeks. The last two weeks, mice were infused subcutaneously with the vehicle (saline, Sal) or oxytocin (Oxt, 50 μg/day). At days 10–13, they were IP injected twice per day (7:00 and 19:00) with saline (days 10–11) and leptin (days 12–13). (**B**–**E**) Cumulative food intake at the indicated timings in the different groups. White bars, saline; black bars, leptin (1.5 mg/kg). Statistical significance was analyzed by two-way repeated measures ANOVA with an FDR Benjamini and Hochberg’s post-hoc test, * *p* < 0.05, ** *p* < 0.01, *** *p* < 0.001.

In the first sets of experiments (Figure 1A), lean (Chow Diet fed, CD) or obese (High Fat Diet fed, HFD) mice were treated subcutaneously for two weeks with oxytocin (Oxt) or its vehicle (saline, Sal) and at the end of the treatment acute intraperitoneal leptin sensitivity was tested. In saline-pre-treated mice, acute leptin administration to lean (CD-Sal) mice reduced food intake (Figure 1B). This leptin effect was absent in obese (HFD-Sal) mice known to be leptin resistant (Figure 1D). In oxytocin-pre-treated mice, leptin reduced food intake in both lean (CD-Oxt, Figure 1C) and obese mice (HFD-Oxt, Figure 1E), where leptin anorexigenic effect was re-established. Further analysis showed that whereas leptin had no impact on meal frequency, it decreased meal duration in all groups, except in HFD-Saline animals that exhibited leptin resistance (Figure S1). In keeping with these results, the leptin effect in decreasing body weight observed in the CD-Sal (−0.99 ± 0.4 g) or the CD-Oxt (−0.88 ± 0.3 g) group disappeared in HFD-Sal (−0.03 ± 0.2 g, *p* < 0.05 vs. CD-Sal), but was restored in HFD-fed mice that were treated with oxytocin (HFD-Oxt, −0.46 ± 0.2 g, *p* = n.s. vs. CD-Oxt) (Figure S2A,B).

**Figure 2:**
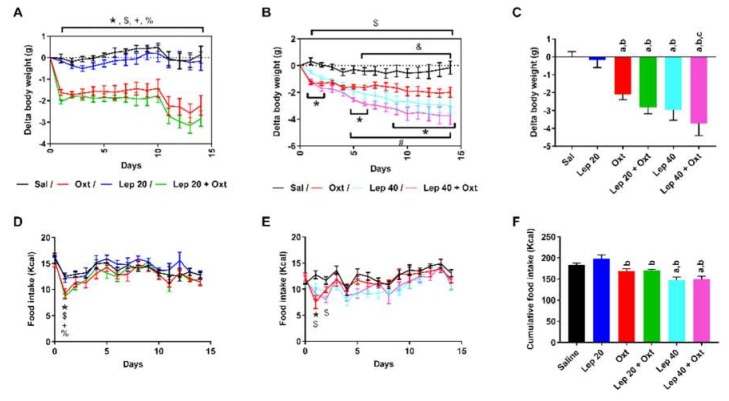
Effects of chronic oxytocin and leptin treatment on body weight and food intake. (**A**,**B**) Delta body weight of HFD mice treated subcutaneously with minipumps infusing saline, oxytocin (50 μg/day), leptin (20 µg/day) (**A**) or (40 µg/day) (**B**), leptin plus oxytocin (20 μg/day and 50 µg/day, respectively) (**A**) or (40 μg/day and 50 µg/day) (**B**). (**C**) Delta body weight at the end of the treatment for the indicated groups. (**D**–**F**) Daily (**D**,**E**) and cumulative (**F**) food intake for the indicated groups. For (**A**,**B**) and (**D**,**E**), statistical significance was analyzed by two way repeated measures ANOVA with a FDR Benjamini and Hochberg’s post-hoc test. For (**C**) and (**F**), statistical significance was analyzed by one-way ANOVA with a FDR Benjamini and Hochberg’s post-hoc test. For (**A**,**B**) and (**D**,**E**): *n* = 6–8, * *p* < 0.05, Sal vs. Oxt; # *p* < 0.05, Sal vs. Lep; $ *p* < 0.05, Sal vs. Lep+Oxt; and *p* < 0.05, Oxt vs. Lep+Oxt; + *p* < 0.05, Lep vs. Lep+Oxt; % *p* < 0.05, Lep vs. Oxt. For (**C**,**F**), *n* = 6–16, a, *p* < 0.05 vs. Sal; b, *p* < 0.05 vs. Lep 20 µg/day; c, *p* < 0.05 vs. oxytocin.

In the second series of experiments (Figure 2), HFD mice were concomitantly treated with leptin and oxytocin administered by continuous subcutaneous infusion. Oxytocin was used at a dose previously reported to reduce body weight gain of genetically obese mice [3], whereas the first leptin dose (20 µg/day) was chosen to be ineffective on any parameter in obese mice (Figure 2A,C,D,F). The combined administration of oxytocin and leptin used at this sub-effective dose had the same impact as oxytocin alone in decreasing body weight (Figure 2A,C). When the amount of leptin was doubled (40 µg/day), it significantly reduced body weight gain (Figure 2B,C). The combination of oxytocin and leptin at this higher dose had the same effect as leptin alone in decreasing body weight (Figure 2B,C). Under these experimental conditions, neither oxytocin, leptin at 20 µg/day, nor oxytocin plus leptin (20 µg/day) had any effect on cumulative food intake (Figure 2D,F). At 40 µg/day, leptin decreased food intake, an effect that was unaltered in the presence of oxytocin (Figure 2F).

None of the treatments used had any effect on the main circulating parameters used to characterize glucose and lipid metabolism (Tables S1 and S2).

## 3. Discussion

Accumulating evidence shows the existence of close interactions between leptin and oxytocin that appear to be important in the regulation of food intake and body weight homeostasis [23,24,25,26].

The aim of the present study was to go one step further by addressing the question of whether chronic (2 weeks) oxytocin treatment of obese mice with hyperleptinemia and leptin resistance could improve leptin sensitivity tested acutely and leptin resistance during chronic leptin infusion.

With regard to the first point, our main observation was that oxytocin treatment of HFD fed mice re-established a normal anorexigenic effect of leptin (Figure 1E), mainly due to an impact of oxytocin on meal duration (Figure S1H). The treatment also partially restored the body weight loss induced by leptin (Figure S2). As suggested by the observation of similar body weight in saline (35.13 ± 0.9 g) and oxytocin infused HFD fed mice (33.25 ± 1.4 g, *p* = 0.27) at the time of the leptin sensitivity test, the beneficial effect of the oxytocin pre-treatment is unlikely related to lower body weight in the oxytocin-treated group. Of additional note, the effects of the oxytocin pre-treatment seen in HFD-fed mice were not observed in lean CD mice. Firstly, lean mice do not exhibit a leptin resistance as is the case for the obese HFD mice and on this scenario, the leptin response is maximal. Secondly, these data are in agreement with previously reported results showing that oxytocin treatment is more efficient in obese, leptin resistant than in lean rodents [3,27]. Finally, we observed that oxytocin pre-treatment did not modify basal food intake in lean or obese mice at the time of the acute leptin sensitivity test (Figure S3 and Figure 2, [3]), not being this parameter responsible for the phenotype observed. Together, these results allowed to conclude that oxytocin treatment of obese mice normalized the acute leptin response.

Given these results, we investigated whether oxytocin treatment would be efficient in improving the effects of chronic leptin administration on food intake and body weight loss of obese mice. This was tested using two different doses of leptin one that was ineffective by itself on both parameters (20 µg/day) and another one that significantly reduced food intake and body weight over 2 weeks of treatment (40 µg/day). The first dose (20 µg/day, equivalent to 0.5 mg/kg) was chosen as it was already reported to decrease body weight in lean mice [28] and to be ineffective in diet-induced obese rats as a monotherapy but showing a synergistic effect when co-administered with amylin [19], whereas the second dose was chosen due to its effectiveness in lean [28] and obese mice (present study). We observed that the effects of oxytocin and leptin co-administration on food intake, body weight, glucose and lipid metabolism (as far as can be judged by the measurements of circulating parameters (Tables S1 and S2)) were not higher than those of leptin or oxytocin alone. This indicated that an oxytocin treatment is unable to improve the effect of chronic leptin administration in HFD-fed mice.

Food intake is regulated by both short and long-term mechanisms. In the study investigating the acute leptin response, one of the possibilities underlying the beneficial oxytocin effects is that oxytocin and leptin would act synergistically to activate Central Nervous System centers involved in the control of food intake [5,29], specifically in the arcuate nucleus, which is the hypothalamic area specifically exhibiting a state of leptin resistance in diet-induced obese rodents [29]. In chronic studies, it is anticipated that the leptin-induced decrease in food intake involves long-term mechanisms. The conclusions that can be drawn from this second experiments are that: (A) Oxytocin-induced body weight loss is mainly food intake-independent as chronic oxytocin treatment decreased body weight without long-term modification of food intake, an observation which likely involves several different mechanisms (reviewed in Reference [1]); (B) the leptin-induced body weight loss depends on both a food intake-dependent and -independent component [30]; (C) considering their effects in decreasing body weight, oxytocin and leptin appear to act on the same effector pathway. Indeed, if they were targeting separate pathways to promote body weight loss, their combination would be expected to exert additional effects. However, the body weight changes measured in response to the efficient dose of leptin (40 µg/day) were not amplified in the presence of oxytocin. Conversely, when leptin was used at a sub-effective dose (20 µg/day), body weight loss induced by oxytocin treatment was maximal and could not be overcome by concomitant leptin administration. Therefore, the results of our second set of experiments suggest that, unlike other hormones such as amylin [20], oxytocin does not modify the long-term effects of leptin. However, further experiments with longer treatments should clearly be carried out to detect a possible occurrence of meaningful improvements in body weight or in glucose or lipid metabolism and investigate the hepatic oxytocin effects, as described elsewhere [3]. Another issue that needs to be addressed is related to the gender, as the present results are limited to male mice, although a previous study concluded that oxytocin treatment of high fat diet obese mice similarly induces body weight reduction in both male and female mice [10].

To conclude, these results suggest that chronic oxytocin treatment of leptin resistant rodents is able to restore a normal acute leptin sensitivity. These observations could be of potential relevance for the treatment of overweight in humans exhibiting leptin resistance.

## 4. Materials and Methods

### 4.1. Mice

Principles of laboratory animal care were followed (European and local government guidelines) and procedures were approved by animal care and experimentation authorities of the Canton of Geneva, Switzerland (animal protocol numbers GE/56/16 authorized from May 2016 and GE/154/16 authorized from December 2016). Eight-week-old C57BL/6JRj male mice (Janvier, France) were fed a high fat (HFD, E15742-34, Ssniff, Germany) or a chow diet (CD, RM3(E)SQC, Special Diets Services, UK) for 10 weeks. The proportion of calories derived from nutrients for HFD was 60% fat, 21% carbohydrate, 19% protein, and 5.73 kcal/g energy density; and for CD 11.5% fat, 61.6% carbohydrate, 26.9% protein, and 3.63 kcal/g energy density. After 8 weeks of CD or HFD, mice were distributed by stratified random allocation, in order to obtain no statistical difference in initial body weight within each dietary group. Basal characteristics of CD and HFD mice are described in Table S3, demonstrating that HFD fed mice gained more weight and presented a diabetic and obese metabolic phenotype.

### 4.2. Leptin Sensitivity Test

The experimental design of the study is schematized by Figure 1A. After 8 weeks of CD or HFD, mice were treated with oxytocin (50 μg/day, PolyPeptide, Strasbourg, France) or the solvent (saline) via subcutaneous minipumps during 2 weeks (Alzet, Cupertino, CA, USA), as previously described [3]. On the 7th day of treatment, mice were transferred into calorimetric cages (TSE, Bad Homburg, Germany), in which food intake was recorded every minute and a leptin sensitivity test was performed as described elsewhere [31]. Then, they were IP injected twice per day (7:00 and 19:00) with saline (vehicle) at days 10th–11th and with leptin (Peprotech, London, UK) at days 12th–13th (1.5 mg/kg). Animals were weighed before each injection. Food intake analysis was performed considering one meal when the feeding recording occurred ≤5 min of the previous one and their sum was ≥0.02 g. If feeding detection was more than 5 min apart, it was considered as a new meal. Meal frequency was the quantity of meals by the mentioned period of time. Meal duration was the time between the first and last recording for each meal and Figure S1 shows the sum of the individual meal durations at the indicated period of time.

### 4.3. Co-Infusion Study

After 8 weeks of HFD, another independent cohort of mice was treated with oxytocin (50 μg/day), leptin (20 µg/day or 40 µg/day) or saline via subcutaneous minipumps, during 2 weeks. Food intake and body weight were measured daily. Osmotic pump content was verified postmortem in order to ensure complete drug delivery.

### 4.4. Biochemical Measurements

At the end of the experiment, after 5 h of fasting (from 10:00 to 15:00, in order to avoid random post-prandial confounding effects), glycemia (Roche, Basel, Switzerland) was measured from tail blood and plasma was obtained from trunk blood. Plasma triglycerides (Human, Magdeburg, Germany), glycerol (Sigma-Aldrich, St Louis, MO, USA), non-esterified fatty acids (Sigma-Aldrich), leptin (Bertin Pharma, Montigny le Bretonneux, France) and insulin (Crystal Chem, Elk Grove Village, IL, USA) levels were measured. HOMA-IR (Homeostasis Model Assessment – Insulin resistance), was calculated as = [fasting insulin (mU/L) × fasting glucose (mM)]/22.5.

### 4.5. Statistical Analysis

Quantitative data are expressed as mean ± SEM. Outlier analysis was performed by ROUT test. Statistical significance was analyzed by (a) Student’s *t* test, (b) one-way ANOVA FDR Benjamini and Hochberg’s post-hoc test or (c) two way repeated measures ANOVA FDR Benjamini and Hochberg’s post-hoc test, when (a) two groups non-longitudinal data, (b) more than two groups non-longitudinal data or (c) longitudinal data was analyzed respectively (each legend contains which test has been used). The false discovery rate (FDR) was kept under 5% and adjusted *p* values for this parameter (also known as *q* values) were shown. Statistical significance was stablished at an adjusted *p*-value lower than 0.05 when FDR control was performed and at *p*-value lower than 0.05 when Student’s *t* test was used. Analysis was performed using Prism (Graphpad, San Diego, CA, USA).

## Supplementary Materials

Supplementary materials can be found at http://www.mdpi.com/1422-0067/20/1/88/s1.
