# Supplementary material for: Oxytocin Administration Alleviates Acute but Not Chronic Leptin Resistance of Diet-Induced Obese Mice
Source: Int J Mol Sci. 2018 Dec 26;20(1):88. doi: 10.3390/ijms20010088 (PMC6337181; doi:10.3390/ijms20010088)
Supplement: Supplementary file 1 [file ijms-20-00088-s001.pdf]

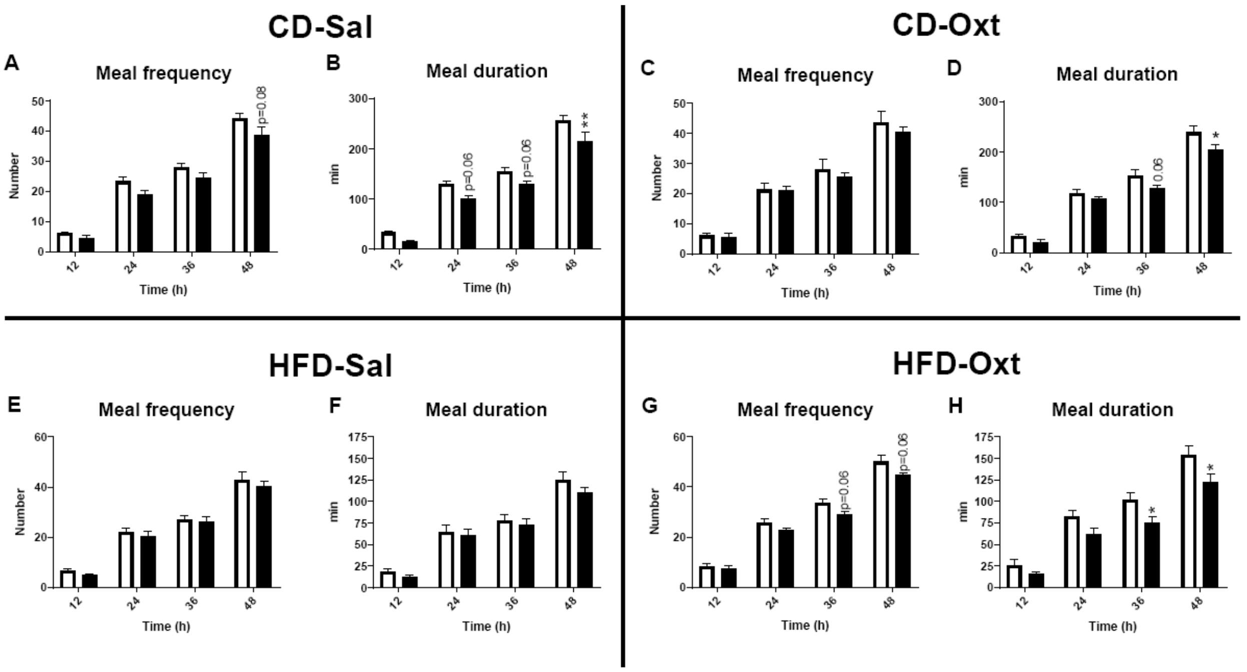

Figure S1. Acute leptin effects, meal analysis. (**A, C, E, G**) Meal frequency and (**B, D, F, H**) meal duration measurements of the acute leptin administration effect at the indicated timings. Statistical significance was analyzed by two way repeated measures ANOVA with a FDR Benjamini and Hochberg's post-hoc test. White bars, saline; black bars, leptin (1.5mg/Kg). n=7-8, * p<0.05, ** p<0.01.

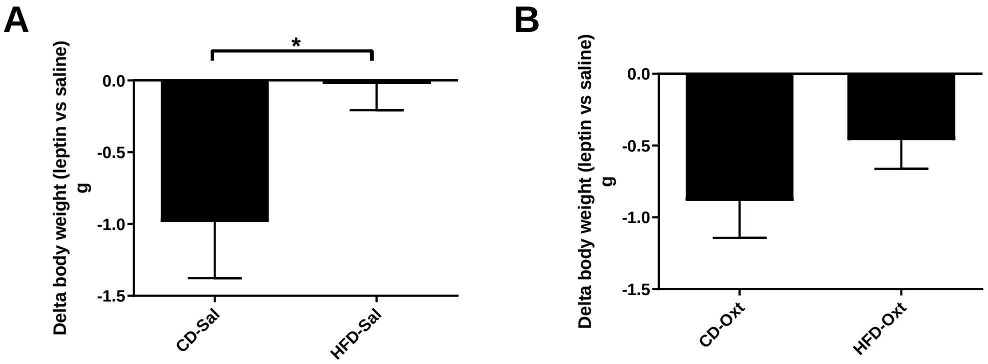

Figure S2. Acute leptin effects on body weight. (**A-B**) Body weight differences between 12 hours after the last IP injection of saline and 12 hours after the last IP injection of leptin in saline (**A**) and oxytocin (**B**)-treated mice. Statistical significance was analyzed by Student's *t* test. n=7-8, * p<0.05.

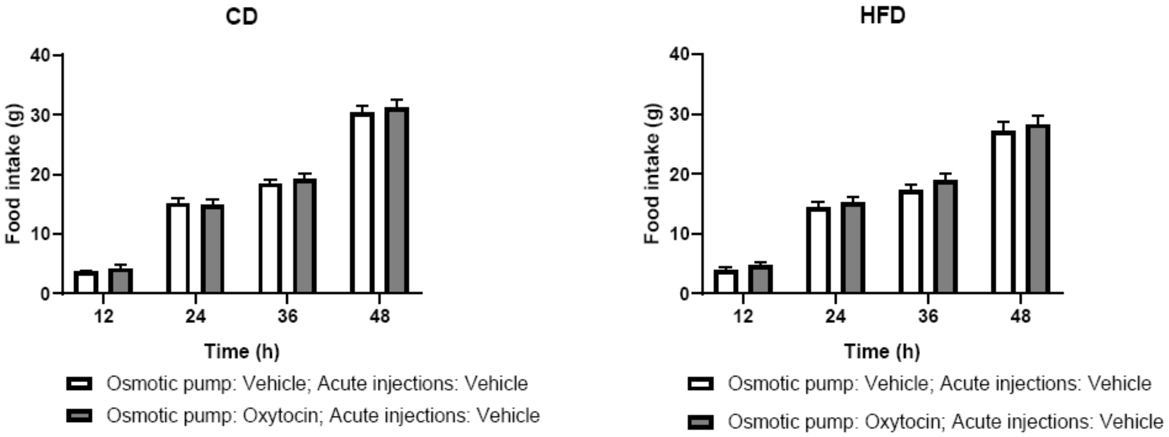

Figure S3. Acute leptin effects, meal analysis. Cumulative food intake at the indicated timings in the different groups. White bars, osmotic pumps delivering saline; grey bars, osmotic pumps delivering oxytocin (50 ug/day). Statistical significance was analyzed by two way repeated measures ANOVA with a FDR Benjamini and Hochberg's post-hoc test.

Table S1. Plasma measurements reflecting glucose (glycemia, insulinemia, HOMA-IR) and lipid (triglycerides, glycerol, non-esterified fatty acids and leptin) metabolism in HFD mice subcutaneously treated with minipumps infusing saline, oxytocin (50 ug/day), leptin (20 μg/day) or leptin plus oxytocin (50 ug/day and 20 μg/day) at the end of the treatment and following removal of food for 5 hours (10h-15h). Statistical significance was analyzed by one way ANOVA with a FDR Benjamini and Hochberg's post-hoc test. n=7-8.

	Sal	Oxt	Lep 20	Lep 20 + Oxt
TG (mg/mL)	0.52 ± 0.06	0.46 ± 0.05	0.54 ± 0.03	0.47 ± 0.03
Glycerol (ug/mL)	86.1 ± 6.7	86.6 ± 5.8	89.5 ± 9.0	89.5 ± 6.9
NEFA (mM)	4.2 ± 0.3	4.5 ± 0.2	4.6 ± 0.2	5.0 ± 0.3
Leptin (ng/mL)	19.1 ± 3.4	14.3 ± 2.1	25.4 ± 3.8	19.2 ± 2.6
Glucose (mM)	9.3 ± 0.4	8.5 ± 0.4	8.6 ± 0.3	8.8 ± 0.3
Insulin (mU/L)	38.9 ± 2.4	41.1 ± 4.5	32.4 ± 1.4	38.8 ± 6.4
HOMA-IR	15.2 ± 1.7	14.5 ± 1.6	12.5 ± 0.8	15.4 ± 2.9

Table S2. Plasma measurements reflecting glucose (glycemia, insulinemia, HOMA-IR) and lipid (triglycerides, glycerol, non-esterified fatty acids and leptin) metabolism in HFD mice subcutaneously treated with minipumps infusing saline, oxytocin (50 ug/day), leptin (40 μg/day) or leptin plus oxytocin (50 ug/day and 40 μg/day) at the end of the treatment and following removal of food for 5 hours (10h-15h). Statistical significance was analyzed by one way ANOVA with a FDR Benjamini and Hochberg's post-hoc test. n=6-8. *, p<0.05 Oxt vs Lep40 + Oxt; †, p<0.05 Oxt vs Lep40.

	Sal	Oxt	Lep 40	Lep 40 + Oxt
TG (mg/mL)	0.88 ± 0.02	0.63 ± 0.02 (*)	0.97 ± 0.12	1.05 ± 0.14
Glycerol (ug/mL)	174 ± 4.6	145.3 ± 5.1 (*, †)	186.8 ± 8.2	188.9 ± 12.1
NEFA (mM)	4.1 ± 0.1	3.8 ± 0.4	4.9 ± 0.3	4.6 ± 0.2
Leptin (ng/mL)	10.1 ± 1.4	11.9 ± 3.2	9.5 ± 1.7	10.4 ± 3.0
Glucose (mM)	10.2 ± 0.2	9.0 ± 0.3	9.5 ± 0.5	9.2 ± 0.3
Insulin (mU/L)	33.3 ± 3.7	30.7 ± 2.1	30.5 ± 2.5	29.8 ± 2.4
HOMA-IR	14.8 ± 1.6	12.1 ± 1.1	12.8 ± 1.3	12.1 ± 1.0

Table S3. Mice basal characteristics. Plasma measurements in CD (chow diet) and HFD (high fat diet) fed mice at beginning of the treatments. Statistical significance was analyzed by Student's *t* test. n=8-15. ** p<0.01 ; *** p<0.001.

	CD	HFD
Body weight (g)	30.88 ± 0.52	35.66 ± 0.90 (***)
Fat content (g)	2.71 ± 0.16	9.70 ± 0.98 (***)
Lean content (g)	25.04 ± 0.52	22.82 ± 0.39 (**)
Glucose (fasting, mM)	7.96 ± 0.29	9.66 ± 0.25 (***)
Insulin (fasting, mU/L)	8.0 ± 0.7	34.9 ± 2.4 (***)
HOMA-IR	2.86 ± 0.34	15.01 ± 1.13 (***)
Leptin (ng/mL)	0.73 ± 0.11	15.27 ± 2.33 (**)
Glycerol (µg/mL)	28.76 ± 1.46	121.9 ± 12.03 (***)
Triglycerides (mg/mL)	0.83 ± 0.08	0.67 ±0.06
Free fatty acids (mM)	0.23 ± 0.02	4.366 ± 0.26 (***)
